# Introduction to the Special Issue: Electrons, water and rice fields: plant response and adaptation to flooding and submergence stress

**DOI:** 10.1093/aobpla/plv078

**Published:** 2015-07-14

**Authors:** Michael B. Jackson, Abdelbagi M. Ismail

**Affiliations:** 1 School of Biological Sciences, University of Bristol, Bristol Life Sciences Building, 24 Tyndall Avenue, Bristol BS8 1TH, UK; 2 International Rice Research Institute, DAPO Box 7777, Manila, Philippines

**Keywords:** Anaerobiosis, low-oxygen stress, rice, upland crops, waterlogging

## Abstract

Flooding and submergence impose widespread and unpredictable environmental stresses on plants and depress the yield of most food crops. The problem is increasing, as is the need for greater food production from an expanding human population. The incompatibility of these opposing trends creates an urgent need to improve crop resilience to flooding in its multifarious forms. This Special Issue brings together research findings from diverse plant species to address the challenge of enhancing adaptation to flooding in major crops and learning from tactics of wetland plants. Here we provide an overview of the articles, with attempts to summarize how recent research results are being used to produce varieties of crop plants with greater flooding tolerance, notably in rice. The progress is considerable and based firmly on molecular and physiological research findings. The article also sets out how next-generation improvements in crop tolerance are likely to be achieved and highlights some of the new research that is guiding the development of improved varieties. The potential for non-model species from the indigenous riparian flora to uncover and explain novel adaptive mechanisms of flooding tolerance that may be introduced into crop species is also explored. The article begins by considering how, despite the essential role of water in sustaining plant life, floodwater can threaten its existence unless appropriate adaptations are present. Central to resolving the contradiction is the distinction between the essential role of cellular water as the source of electrons and protons used to build and operate the plant after combining with CO_2_ and O_2_ and the damaging role of extracellular water that, in excess, interferes with the union of these gases with photosynthetic or respiratory electrons and protons.

## Abstract (precis)

Flooding and submergence impose unpredictable environmental stresses, considerably reducing the yields of most crops. Efforts to improve crop resilience to flooding will help in enhancing and sustaining crop production to cope with increased demands for food. This article provides an overview of the papers presented in the Special Issue “Plant Responses to Low Oxygen Environments”, summarizing recent research results and how it is guiding the development of flood-tolerant crops and varieties. The potential for exploiting non-model wetland species to uncover and explain novel adaptive mechanisms of flooding tolerance that may be introduced into crop species is discussed.

## Introduction

The threat to plants, especially to crop plants, posed by flooding of the soil or from deeper submergence, is a global concern that increases year by year. It arises from a cluster of related and convergent worries. For example, many areas of the world are experiencing or are predicted to experience increasingly erratic weather patterns that include more frequent and lengthy storms that flood farmland (Christensen and Christensen 2003; Jongman *et al*. 2014; Shi and Tao 2014). Millions of dollars worth of annual losses in crop yield over the past 25 years or so, caused by flooding, have been documented for various parts of the world, including the USA, China, Europe and Australia (Bailey-Serres *et al*. 2012; Shi and Tao 2014). These losses occur in the context of a requirement for greater crop production as the human population expands (Website 1) and demands for a western-style diet continue to grow. To these concerns can be added the limited scope for expanding the area for cultivation, especially if what is left of our dwindling natural forests and wetlands are to be conserved (Fischer and Heilig 1997). The threat of rising sea levels adds the further concern that increased flooding in coastal plains, estuaries and deltas will reduce agricultural productivity in these presently fertile areas, sometimes in combination with seawater salinization (Chen *et al*. 2012).

**Figure 1.**
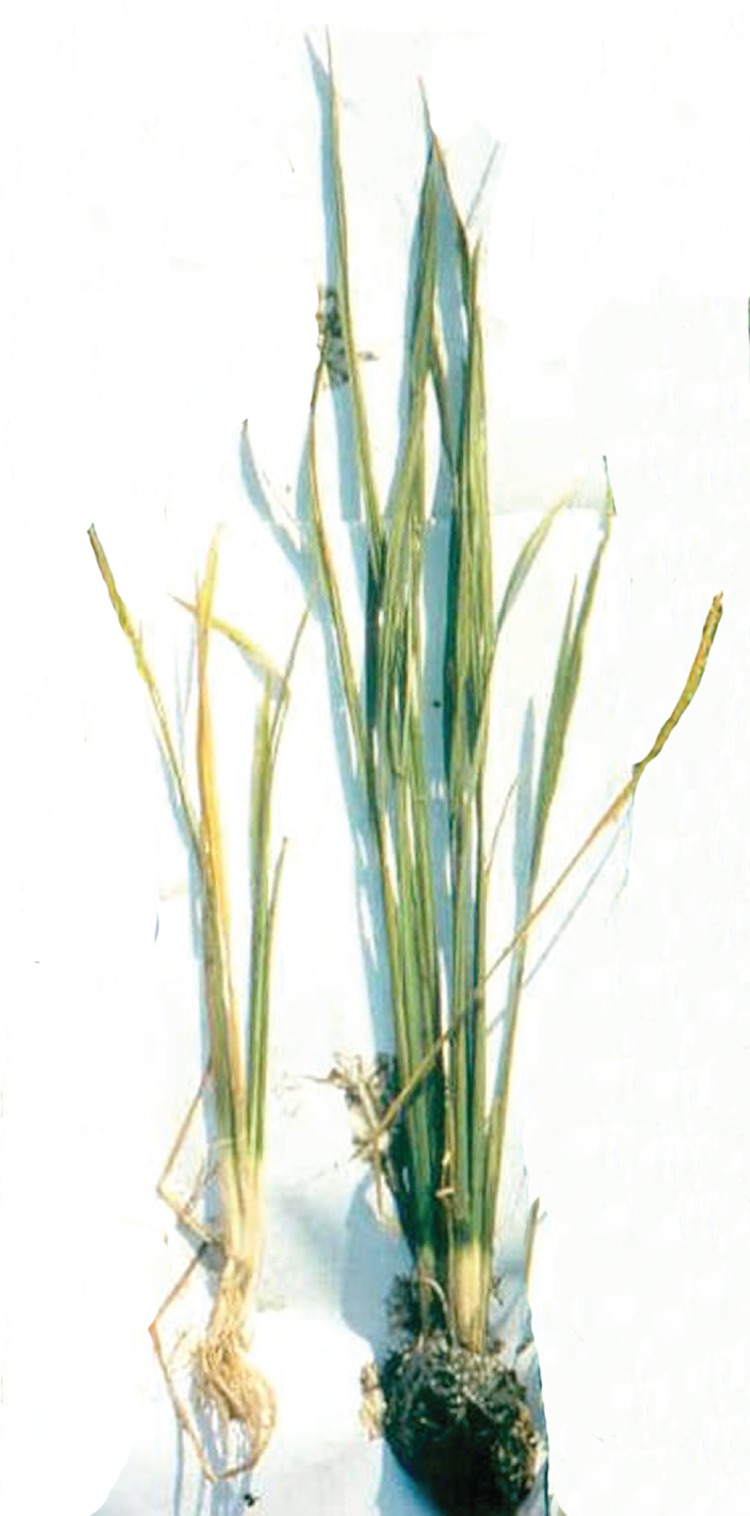
Damage to a deepwater cultivar of rice caused by total submergence under field conditions. Left: submerged for 7 days before de-submerging for a further 14 days. Right: grown throughout with most leaves maintained at least partially above water. Photograph by Dr Panatda Bhekasut (Prachinburi Rice Research Center, Bansang, Thailand).

**Figure 2.**
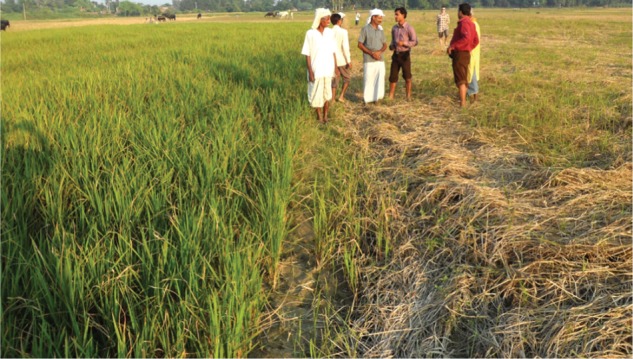
Improving tolerance of rice to complete submergence based on molecular technology that identified a locus (*SUB1*) on Chromosome 9 of a tolerant farmer old variety (FR13A) that adopts a quiescence strategy to minimize the injury. Right: standard farmer variety severely affected by 12 days of complete submergence in Uttar Pradesh, India, in 2012. Left: mega-variety Swarma with the *SUB1* locus introgressed by crossing with an FR13A derivative. Subsequent marker-assisted selection produced a highly submergence-tolerant cultivar that retained the desirable agronomic qualities of the original.

These trends have been recognized for many years in relation to flooding and to other environmental stresses (Jackson 1993) and this has spawned a sizeable international research effort into mechanisms of stress response and tolerance. The ‘Plantstress’ web site gives an excellent overall picture (Website 2). Research on flooding stress, in particular, has yielded results of practical value that are based firmly on a fast-growing body of research at molecular, whole plant and crop levels (Visser *et al*. 2003; Jackson and Colmer 2005; Jackson *et al*. 2009; Perata *et al*. 2011; Bailey-Serres and Colmer 2014; Colmer *et al*. 2014). Even rice, well known for its ability to yield heavily in monsoon conditions, is not immune from injury (Fig. 1) or crop failure as a result of uncontrolled flooding or submergence. However, spectacular progress in understanding the genetic and molecular regulation of responses and adaptation by rice (Xu and Mackill 1996; Nandi *et al*. 1997; Xu *et al*. 2006; Angaji *et al*. 2010) has bolstered plant breeding work that has given us new cultivars of rice with agriculturally desirable levels of tolerance to total submergence (Fig. 2) (Siangliw *et al*. 2003; Neeraja *et al*. 2007; Septiningsih *et al*. 2009). Seeds of the latest ‘waterproof’ cultivars are now available at prices farmers in rainfed lowlands in Asia can afford and on a scale that is sufficiently large to have a wide and long-lasting impact (Mackill *et al*. 2012; Ismail *et al*. 2013). In this article, we provide an overview and summaries of the papers presented in this Special Issue, highlighting recent findings on plant adaptation to flooding and potential application for breeding flood-tolerant crops.

## How Are Plants Damaged by Flooding and Submergence?

With so much concentration on mechanisms that enhance resilience to flooding and submergence and the utilization of this information to improve crop tolerance, it is instructive to remind ourselves of the basic mechanisms by which flooding threatens plant life. After all, water itself is chemically benign and makes up the bulk of healthy growing tissues. How a foundation molecule such as this can injure or kill is a seeming conundrum. An important starting point to resolving the contradiction is recognizing that that problem comes not from internal water but from excess water that lies outside the plant. How this extracellular water puts underlying tissues at increasing risk as the depth of water cover increases above ∼1 mm (Jackson 1979) is summarized briefly below in terms of blocking the electron flows that underpin photosynthesis and aerobic respiration. As every biology student is taught, cellular water is the source of these electrons. In photosynthesis, these electrons, along with protons (also from water), are passed to CO_2_, the energy and chemical reducing power this creates then being used to form the sugars (Malkin and Niyogi 2002) used in subsequent anabolic and catabolic biochemistry. The electrons are, initially, released by oxidation of water when photons oxidize chlorophyll stepwise until it acquires sufficient positive charge to cleave water molecules. The resulting sugars are therefore rich in water-sourced electrons, which are released during subsequent aerobic mitochondrial respiration and passed step by step back to O_2_ through a complex set of chemical reactions to recreate water while generating readily useable metabolic energy (ATP) (Siedow and Day 2002). Photosynthetic and respiratory electron flows thus make land-based (i.e. aerobic) plant life energetically possible, with the ATP formed being used in numerous essential processes including cell maintenance and the re-organization of remaining unrespired sugars to form reserves and the structural and functional mass of the cell.

These key energy-transferring electron flows terminate either in CO_2_ or in O_2_. However, an external water covering frustrates the plant's addiction to these gases by inhibiting the rate at which they feed by diffusion into the plant from the air across the water barrier. The slowing effect is in the order of 10 000 (Armstrong 1979). Without an ample supply of either gas, electron flows are arrested and the plant suffers energy shortfalls that can prove fatal. Higher plants are therefore obligate consumers of both CO_2_ and of O_2_ with flooding interfering with their entry into respiring or photosynthesizing cells.

The inward fluxes of CO_2_ and O_2_ required to keep electrons flowing normally are considerable. Photosynthetic influx of CO_2_ can be in the order of 350 μmol kg^−1^ s^−1^ and respiratory demands of roots for O_2_ are ∼50 μmol kg^−1^ s^−1^ (Armstrong *et al*. 1991; Poorter *et al*. 1991; Haupt-Herting and Fock 2002). There can be some mitigating factors. On the demand side, gas requirements per unit absorptive surface area can be minimized by presenting large surface to volume ratios (i.e. thin roots or leaves), cooler temperatures and possibly adaptive metabolic down-regulation of oxygen demand. On the supply side, surface films of trapped gas or thin water layers lessen barriers to the ingress of atmospheric O_2_ and CO_2_. In addition, well-stirred water in equilibrium with air can supply more of these gases (especially O_2_) by convective mass flow (the basis of successful plant solution culture), thereby circumventing a total dependence on diffusive supply. Moving water also thins the unstirred layers that inevitably cling to leaves and roots, thereby maximizing the diffusive gradient driving the gases into the plant from its surface. For O_2_, its modest solubility in water reduces the maximum possible concentration at the plant surface to 0.25 mol m^−3^, well below the 8.31 mol m^−3^ present in free air (at 25 °C). This decreases considerably the concentration gradient driving O_2_ entry via diffusion. A compensation is the high concentration of O_2_ present in air (∼20.8 % by volume). A second compensation is the strong affinity for O_2_ by cytochrome oxidase at the final electron-donating step in respiratory electron transport (*K*_m_ = ∼0.14 mmol m^−3^). This means that an almost total arrest of O_2_ influx is needed to induce fatality in, for example, young elongating cereal roots (Blackwell and Wells 1983).

The situation for CO_2_ is somewhat different. Although CO_2_ is much more soluble in water than O_2_, the much smaller amounts of CO_2_ in air (∼360 ppm, v/v) compared with O_2_ mean that a maximum amount of dissolved CO_2_ concentration in water in equilibrium with air is ∼2000 times below that of O_2_ in the same water. Photosynthesis is thus more at risk from CO_2_ starvation caused by flooding than respiration is from O_2_ starvation. Photosynthetic electron transport is also put at risk by the relatively low affinity for CO_2_ of Rubisco, the enzyme largely responsible for capturing photosynthetic CO_2_ (*K*_m_ for Rubisco—10 mmol m^−3^ compared with 0.14 mmol m^−3^ for O_2_ and cytochrome oxidase). This means that, although roots can respire normally in water equilibrated with air, it is not possible for submerged leaves to photosynthesize normally under these same conditions. An artificial supplement of CO_2_ is needed to achieve this. This was demonstrated by Setter *et al*. (1989) who grew rice plants to full size over many weeks when completely submerged but only if the water was highly enriched with ∼10 % v/v CO_2_. Sparging the water with normal air, which contains only ∼360 ppm CO_2_, was not effective. There are several further challenges to plant life posed by flooding and submergence. These include temperature and shading effects of water especially if it is turbid, the buffeting effects of fast-flowing water and the potentially toxic effects of soil nitrate, iron and manganese when transformed to nitrite, ferrous and manganous ions by the respiratory electron flows of anaerobic soil bacteria. Other soluble metals can also be damaging in flooded soils (Das *et al*. 2009; Setter *et al*. 2009; Kirk *et al*. 2014).

In these various ways, flooding and submergence pose considerable challenges for plant growth and survival. But, within limits imposed by depth, duration, timing and intensity, they can, in some species, be overcome by appropriate combinations of morphological and biochemical attributes or adaptations. The existence of a thriving marshland and riparian flora gives ample illustration of this, although many other species, perhaps all, have some semblance of resilience.

The present article introduces a special issue of *AoB PLANTS* entitled ‘Plant Responses to Low-Oxygen Environments’ (http://aobpla.oxfordjournals.org/cgi/collection/plant_responses_to_low-oxygen_environments). Its papers explore the effects of flooding and submergence, and examine possibilities for improving tolerance to various kinds of flooding stress, notably in rice. The articles are by authors who contributed to the 11th triennial conference of the International Society for Plant Anaerobiosis (Website 3) held at the International Rice Research Institute (IRRI), Los Baños, the Philippines in October 2013. The conference attracted over 130 participants from 30 countries. A second set of articles based on the conference was published in *Plant Cell and Environment* in 2014 (Vol. 37, pp. 2211–2452).

## Topics Discussed in the Special Issue

The 13 papers in the Special Issue address the responses of diverse plant species to various types of flooding and low-oxygen stress. Its contents are summarized below.

### Morphological and anatomical adaptations to low-oxygen environments

Several decades of study have highlighted the importance of inherent and induced growth and anatomical re-tuning necessary for successful plant adaptation to flooded conditions and the associated shortages of oxygen or CO_2_. Examples of these traits include (i) constitutive and/or induced enhancement of tissue porosity (aerenchyma) to transmit oxygen from aerial parts to roots and other inundated plant organs; (ii) constitutively large carbohydrate reserves; (iii) an ability to assimilate carbon underwater to fuel growth or (iv) sustain anaerobic respiration when internal oxygenation is hindered (Jackson and Armstrong 1999; Colmer 2003; Ismail *et al*. 2009). Fast extension growth by leaves or stems to restore contact with air before submerged plants asphyxiate is an important escape mechanism in numerous species and is driven hormonally by entrapped ethylene or by a lack of oxygen *per se*, depending on the species or organ (Summers and Jackson 1994). When flooding is deep but transient, mechanisms that suppress underwater shoot elongation (Bailey-Serres and Voesenek 2008; Bailey-Serres *et al*. 2010) can lead to increased survival by slowing the consumption of respirable substrates that are then available for maintenance and recovery growth once floodwater recedes.

In response to the quandary of increasing demand for food from less reliable and deteriorating resources, **Voesenek, van Veen and Sasidharan** (2014) argue for extending research efforts to non-model crops to disclose novel adaptive mechanisms. Work on species that have evolved in habitats naturally affected by recurrent floods such as *Rumex* and *Rorippa* spp. could, potentially, provide useful knowledge and resources for breeding crop plants with enhanced tolerance of flooding or low-oxygen stress. Several of the articles presented in this special issue support this argument. For example, **Cardoso, Jiménez and Rao** (2014) observe that *Brachiaria humidicola*, an important stoloniferous forage crop in the acid soils of Latin America, adapts to waterlogged conditions by restricting root growth to the top 30 cm and increasing the proportion of lateral roots (originating from nodal roots) in the top 10 cm of the soil. Lateral roots are less able to form aerenchyma when flooded than main axis roots. The authors argue that this could compensate for the loss of absorptive root surface resulting from restricted growth at depth. Additionally, this may also reduce the overall demand for oxygen by lateral root tips.

A second non-model species, *Solanum dulcamara*, forms dormant root primordia on stems constitutively. **Dawood, Rieu, Wolters-Arts, Derksen, Mariani and Visser** (2014) find that these root primordia commence synchronized growth to form roots within 2–3 days of flooding through a combination of cell division and elongation. However, expression of ethylene- and hypoxia-related genes starts within 2 h of flooding. These adventitious roots contain aerenchyma tissue and could functionally replace primary roots that usually disintegrate during flooding, hence aiding survival and growth (Sauter 2013; Yamauchi *et al*. 2013).

**Huber, Visser, Clements and Peters** (2014) examine the effects of flooding and stem fragment size on survival and regrowth of two contrasting stoloniferous species, *Trifolium repens*, a common inhabitant of highly disturbed riverine grasslands and *T. fragiferum* dominating mostly in coastal dunes. The authors report reductions in survival and regrowth of fragments sprouted after flooding in both species, but with a smaller effect on clones collected from riverine grasslands compared with coastal dunes. Ramets attached to larger internodes were expected to benefit from the larger energy reserves but, unexpectedly, this was not the case for survival, though regrowth of surviving plants does benefit from being attached to larger internodes. The authors attribute this to selection pressures on internode size in stoloniferous species growing in severely disturbed habitats.

Studies such as these have examined relatively neglected wild species. The results clearly show that species adapted to aquatic and flood-prone areas have developed their own unique combination of tactics to help them endure low-oxygen stress. Potentially, these could be exploited in future breeding programmes to develop tolerant crop varieties for waterlogged and flood-affected areas.

### Metabolic adaptation to flooding

Plants have evolved metabolic means to help survive in areas affected by flooding. These include shifting from aerobic to fermentative pathways to acquire energy from stored reserves in the absence of oxygen. **Estioko, Miro, Baltazar, Merca, Ismail and Johnson** (2014) evaluate two rice genotypes of contrasting tolerance of flooding whilst germinating. They also include two barnyard grasses *Echinochloa crus-galis* and *E. colona* well known as serious paddy field weeds. The study demonstrates that rice is more flood-tolerant than either of the barnyard grass weeds, a difference associated with the stronger expression of the two main fermentative enzymes—pyruvate decarboxylase (PDC) and alcohol dehydrogenase (ADH). These are differentially enhanced in rice with higher activity in more tolerant genotypes but with similar and slower up-regulation in the barnyard grass weeds. On the other hand, activity of aldehyde dehydrogenase (ALDH) is up-regulated only in the more tolerant rice genotypes or in barnyard grass, implying that ALDH is important in both species to detoxify acetaldehyde generated during fermentation. The induction was much greater in rice. The work also finds that, although flooding to a depth of 10 cm water suppresses both weed species, it has little effect on a tolerant rice genotype. Studies like this provide basic information needed to develop effective weed control measures for crops such as rice.

Aerenchyma, the interconnected tissue that allows internal transport of oxygen and other gases within the plant tissue, is a common characteristic in most plants adapted to flooded habitats (e.g. Justin and Armstrong 1987). Numerous species, such as rice, constitutively develop aerenchyma tissue in their roots, though this can be enhanced further by flooding and ethylene. The role of ethylene in inducing aerenchyma formation under flooded, yet hypoxic conditions has long been established; however, its role in constitutive aerenchyma formation taking place under well-aerated conditions has not been much studied. **Yukiyoshi and Karahara** (2014) describe a novel sandwich system enabling them to manipulate ethylene synthesis and sensitivity on developing rice roots, either on one side or on both sides of individual root axes. Their studies indicate the necessity of endogenous ethylene in signalling constitutive aerenchyma formation.

Rice is the only major crop species with seeds capable of germination under anaerobic conditions (see Ismail *et al*. 2009; Magneschi and Perata 2009). It is an example of an escape mechanism based on organ elongation that is promoted by the absence of oxygen. Although the molecular mechanisms enabling this remain largely unknown, they are starting to be uncovered by work combining *in silico* modelling with gene expression data analyses. Using this approach, **Lakshmanan, Mohanty, Lim, Ha and Lee** (2014) identify pathways that are differentially regulated during germination when oxygen is limiting and are likely to be associated with survival under low-oxygen stress. They show genes linked to sucrose catabolism and fermentation pathways being up-regulated under anaerobic conditions while others controlling central processes that run aerobically, such as oxidative phosphorylation, tricarboxylic acid cycle and pentose phosphate pathway, being down-regulated. Furthermore, the study identifies 37 reactions from rice central metabolism that are transcriptionally regulated. Several *cis*-acting regulatory elements are identified that may regulate functions of transcription factors associated with anaerobic responses. These factors include MYB, bZIP, ERF and ZnF. Future studies of this kind will help to uncover the molecular control mechanisms underlying the functions of these factors in supporting anoxic germination and coleoptile elongation by rice.

### Tolerance of partial and complete submergence in rice and mechanisms associated with tolerance

In rainfed lowlands, water stagnates in paddy fields at 25–50 cm for most of the season. This type of flooding, commonly referred to as ‘stagnant flood’ (SF) or ‘medium-deep’, reduces grain yield over millions of hectares in Asia and Africa. Farmers there are forced to use low-yielding old farmer varieties and landraces because modern varieties are overly sensitive to this kind of flooding. Two articles, **Vergara, Nugraha, Esguerra, Mackill and Ismail** (2014) and **Kato, Collard, Septiningsih and Ismail** (2014), focus on the stress from stagnant flooding. **Vergara, Nugraha, Esguerra, Mackill and Ismail** (2014) describe a unique screening protocol used to evaluate over 600 rice accessions for tolerant genotypes. The work establishes that varieties suitable for areas prone to stagnant flooding possess a moderate elongation rate when submerged. Lines that are semi-dwarf under well-drained conditions but elongate quickly underwater do not perform well under SF. The moderately elongating accessions identified in this study will be used for breeding SF-tolerant varieties. The study of **Kato, Collard, Septiningsih and Ismail** (2014) focuses on traits associated with tolerance of SF amongst contrasting genotypes. It highlights the severe loss of grain yield that SF brings about, even when plants are not totally submerged; the severe impact of SF being attributable to a severe reduction in biomass, reduced leaf area, less light interception and reduced tillering. The latter effect causes >50 % reduction in the numbers of grain-containing panicles per unit land area. Both these studies of SF indicate that, in order to yield well under these conditions, varieties should possess moderate elongation potential of 1.5–2 cm per day during vegetative shoot growth while retaining the ability to produce tillers that bear large fertile panicles and produce sizeable photosythesizing biomass above water. These studies recognize the large gains that could be made by combining tolerance of SF with tolerance to transient complete submergence (*SUB1* type). Such a combination of tolerance traits holds much promise for most flood-prone areas of Asia, where complete submergence usually precedes or succeeds SF (Singh *et al*. 2011).

In deepwater (DW) regions, depth of the floodwater is frequently greater than 50 cm and can reach several metres at times. Rice varieties adapted to these conditions have developed the striking ability to increase the rate of stem internode elongation as the water rises. This helps to maintain contact with air and avoid suffocation by total submergence. This rapid elongation is promoted by ethylene and gibberellic acid (GA) preceded by an enabling drop in the concentration of the elongation inhibitor abscisic acid (ABA). Several studies identified QTLs associated with internodal elongation in DW rice (Sripongpangkul *et al*. 2000; Kawano *et al*. 2008; Hattori *et al*. 2009) and recently, a large-effect QTL on chromosome 12 was identified as the major determinant of rapid internode elongation. Cloning of this QTL revealed two functional genes, *SNORKEL1* (*SK1*) and *SNORKEL2* (*SK2*), that are responsible for this rapid internode elongation. Past studies established the role of GA in internode elongation in DW rice (Raskin and Kende 1984; Hattori *et al*. 2009), but genes that mediate the response to GA have not, hitherto, been identified. This is investigated by **Nagai, Kondo, Kitaoka, Noda, Kuroha, Angeles-Shim, Yasui, Yoshimura and Ashikari** (2014). They carried out an elegant set of trials using a set of DW-tolerant and DW-sensitive genotypes and also recombinant inbred lines developed from their intercrosses. The authors identify two major QTLs on chromosomes 3 and 9, associated with GA-induced internode elongation. They further establish that the QTL on chromosome 3 enhances the effect of other QTLs. Identifying the specific genes associated with these QTLs will further our understanding of the mechanisms of fast underwater internode elongation in rice, with consequential implications for breeding improved DW cultivars.

Although floods that submerge most but not the entire shoot are common, rice in rainfed lowlands is also subject to transient flash-floods that completely submerge the plant, especially small plants, for periods from few days to over 2 weeks. This devastates millions of hectares of rice fields in Asia each year (Setter *et al*. 1997; Jackson and Ram 2003). The recent cloning of the *SUB1A* gene facilitated the development of functional markers that were effectively used for its transfer into several modern varieties using marker-assisted back-crossing. These varieties were subsequently deployed to farmers in flood-prone areas of Asia (Xu *et al*. 2006; Septiningsih *et al*. 2009). *SUB1* can provide protection against up to 2 weeks of complete submergence, with considerable yield benefits (Mackill *et al*. 2012; Ismail *et al*. 2013). However, in recent years major rice producing countries have suffered from floods lasting more than 20 days, suggesting the need for tolerance to more prolonged total inundation. To assess the growth and survival processes conferred by the *SUB1A* gene and potential for improving tolerance with additional traits, **Singh, Mackill and Ismail** (2014) evaluate a set of Sub1 near isogenic lines together with the donor of the *SUB1* QTL (FR13A) and two of its immediate derivatives. Genotypes possessing *SUB1* show better regulation of non-structural carbohydrates during submergence and restore leaf and tiller formation faster during recovery, resulting in higher yields. However, the *SUB1A*-donating cultivar FR13A shows higher tolerance than intolerant varieties that are introgressed with *SUB1* alone. The superiority of FR13A is linked with higher carbohydrate stored in stems before submergence and slower leaf elongation when submerged; but also to higher percentage survival and faster recovery afterwards. The study clearly demonstrates the possibilities for improving submergence tolerance in rice beyond that conferred by *SUB1* alone, and highlights the need for further studies to identify additional genes present in FR13A that complement *SUB1A* effects.

### Tolerance of waterlogging in dryland crops

Dryland crops such as legumes, cereals and non-food crops like cotton are sensitive to soil waterlogging at all stages. **Malik, Ailewe and Erskine** (2015) evaluate waterlogging tolerance in three dryland leguemes—pea, lentil and grasspea. Their studies show a genetic variation between the three species in response to waterlogging during vegetative growth with grasspea (*Lathyrus sativus*) being the most tolerant. The genetic variation was also observed within lentil and pea genotypes although many more genotypes need evaluating. Cotton is one of the most important fibre and oilseed crops grown on over 30 million hectares worldwide. Most cotton areas are heavy clay soils with poor drainage, subjecting the crop to occasional waterlogging causing significant yield losses; yet, studies on responses of cotton to waterlogging are surprisingly scant. **Najeeb, Bange, Tan and Atwell** (2015) provide insights into the causes of waterlogging-induced losses in yield and review the current knowledge on adaptive mechanisms in cotton. They further suggest remediation strategies through combinations of germplasm improvement based on knowledge from other plant species, and management, including nutrient manipulation. The use of plant growth regulators such as anti-ethylene agents can reduce young fruit abscission caused by waterlogging. The authors highlight gaps in research on the genetics, physiological and biochemical responses likely associated with waterlogging tolerance, as targets for future studies in cotton. Selecting suitable crop species and breeding-tolerant genotypes within species coupled with proper management and remedies could, therefore, enhance adaptation and improve productivity of drylands prone to waterlogging.

## Concluding Remarks and Future Directions

There is little doubt that the ever-increasing analytical power of molecular genetics (e.g. gene expression studies, cloning and next-generation genome sequencing) will continue to underpin our understanding of response and adaptation to flooding. The adaptations can, broadly speaking, be seen in terms of either enhancing resilience through quiescence (e.g. down-regulation of core metabolic genes) or by promoting an escape from asphyxiation by means of pre-existing morphological features (e.g. aerenchyma) or from their rapid acquisition in the face of stress (e.g. accelerated underwater shoot elongation) (Jackson 2006). Much is being learned about the transcriptional regulation of the key processes involved (Voesenek and Bailey-Serres 2015). These include the sensing of oxygen shortage, activation or repression of hypoxia-responsive genes, stimulating or suppressing cell elongation and the formation of lysigenous aerenchyma by means of spatially directed programmed cell death (Yamauchi *et al*. 2013). Identifying genes and gene–product interactions involved in these and other adaptive events should open up opportunities for improving resilience to flooding in crop species by genetically manipulating the intracellular pathways that influence them (Voesenek and Bailey-Serres 2015). This is a challenge not to be underestimated. Work to match the molecular analysis to each adaptation and marrying them together into an effective genetic recombination has hardly begun (Peña-Castro *et al*. 2011). The problems are especially challenging when the ambition is to move adaptive features between species so that the attributes of non-model or non-crop plants can be effectively exploited. The creation or exploitation of existing collections of wheat/non-wheat amphiploids represents one long-established approach that may yet yield practical benefit, since they offer means of linking physiological parameters that influence flooding tolerance with readily identifiable chromosome segments of wild relatives with desirable traits (Malik *et al*. 2011).

Flooding stress comes in many guises, each one presenting the plant with a different set of problems requiring a combination of adaptations. For example, waterlogging of the soil risks asphyxiating roots through oxygen shortage, with knock-on damage to the shoot systems that depend on them. Root survival is favoured by the presence of an extensive aerenchyma, as in rice (Yamauchi *et al.* 2013). But to be successful, it must presumably be allied to mechanisms minimizing radial loss of internal oxygen (Shiono *et al*. 2014). It will also need allying to hypoxia-induced root apex quiescence and the ability of the apex to grow with renewed vigour once fully re-aerated, as in aerenchymatous willow (*Salix alba*) (Jackson and Attwood 1996). Results of crossing highly aerenchymatous tiosinte maize (*Zea nicaraguensis*) with cultivated maize (*Z. mays*) show that increasing aerenchyma alone is simply not enough to confer waterlogging tolerance (Mano and Omori 2013). The knowledge gained in efforts to overcoming these challenges will be especially useful in the even more complex but pressing question of improving tolerance of crops to the combined stresses of flooding and salinity. The increasing threat from coastal flooding and salinity interactions with waterlogging of much fertile agricultural land as sea levels continue to rise (Hoggart *et al*. 2014) make such work a priority for the future.

So far, the most notable progress in improving flooding tolerance has been made with transient submergence tolerance in rice. The success has come from stepwise advances over more than 30 years in which wide phenotypic screening for tolerance traits was followed by physiological analysis, fine linkage mapping and QTL analysis. The latter led on to conventional crossing, back-crossing, refined marker-based selection and tolerance selection, bulk seed production and widespread distribution to Asian farmers (Jackson and Ram 2003; Ismail *et al*. 2013). This approach neatly side-steps the opposition to food crops modified by recombinant DNA technologies that prevails many countries. The use of such procedures will continue to help increase resilience to different types of flooding stress in rice and other species and identify the genes involved (Osman *et al*. 2013). The papers in this special issue point to how achieving these gains is progressing and indicate certain adaptive characteristics in non-crop species that could be harnessed in the future while also being of botanical interest in their own right.

## Contributions by the Authors

Both authors contributed equally to this article.

## Conflict of Interest Statement

None declared.
